# Indirect measurement of research misconduct among Iranian postgraduate students of medical sciences using the unmatched count technique

**DOI:** 10.1186/s41073-026-00202-5

**Published:** 2026-06-28

**Authors:** Sanaz Omidi, Younes Mohammadi, Fariba Fatehi, Abouzar Raeisvandi, Fatemeh Ebrahimi, Tina Lari, Hanieh Moradi, Marzieh Otogara, Masoumeh Javaheri

**Affiliations:** 1 Department of Epidemiology, School of Public Health, Hamadan University of Medical Sciences, Hamadan, Iran; 2 Modeling of Noncommunicable Diseases Research Center, Institute of Health Sciences and Technology, Hamadan University of Medical Sciences, Hamadan, Iran; 3 Vice Chancellor for Research and Technology, Kurdistan University of Medical Sciences, Sanandaj, Iran; 4 Department of Epidemiology and Biostatistics, School of Public Health, Isfahan University of Medical Sciences, Isfahan, Iran; 5 Department of Epidemiology, School of Public Health, Iran University of Medical Sciences, Tehran, Iran; 6 Non-Communicable Diseases Research Center, Research Institute for Prevention of Non-Communicable Diseases, Qazvin University of Medical Sciences, Qazvin, Iran; 7 Department of Community Medicine, School of Medicine, Hamadan University of Medical Sciences, Hamadan, Iran

**Keywords:** Research Misconduct, Medical Students, Ethics, Research, Cross-Sectional Study, Iran

## Abstract

**Background:**

Research misconduct poses a serious threat to academic integrity, particularly in medical sciences. This study aimed to estimate the prevalence of various forms of research misconduct include plagiarism, data fabrication or falsification, authorship misconduct, salami slicing, and purchasing research work among postgraduate students in Iranian medical universities using the Unmatched Count Technique (UCT).

**Methods:**

A cross-sectional survey was conducted among postgraduate students from multiple Iranian medical universities using a double-list version of the unmatched count technique (UCT). The questionnaire was administered in two sequential waves, with approximately half of participants completing List A and the remaining participants completing List B, ensuring that each respondent received only one list version. For each research misconduct behavior, prevalence was estimated by calculating the mean difference in endorsement counts between lists containing the sensitive item and corresponding control lists with only non-sensitive items. In the double-list design, prevalence estimates were computed separately for List A and List B, with sensitive items counterbalanced across list positions to control for order effects. The final prevalence was calculated as the average of the two list-specific estimates, improving precision and reducing list-order bias.

**Results:**

The most commonly reported misconduct was using others’ ideas or phrases without proper citation (43%), followed by dishonest result reporting (38%), data fabrication or deletion (34%), and authorship misrepresentation (34%). Salami slicing was reported by 26%, and 20% admitted to purchasing parts or all of a research project. The UCT survey tool demonstrated acceptable reliability, with intraclass correlation coefficients (ICCs) ranging from 0.64 to 0.84.

**Conclusion:**

The findings indicate a troubling level of research misconduct among postgraduatestudents in Iran’s medical sciences universities. This highlights the need for effective ethics training, stronger academic integrity policies, and enforceable institutional mechanisms to promote responsible research conduct and protect the future credibility of medical professionals.

**Supplementary Information:**

The online version contains supplementary material available at 10.1186/s41073-026-00202-5.

## Introduction

Research misconduct has emerged as a global and pressing challenge across numerous scientific disciplines, with particularly serious implications in the biomedical sciences. Although institutions have made efforts to prevent and detect unethical research practices, evidence suggests that the true prevalence of misconduct remains substantially underreported [1, 2], and projections indicate that such behaviors are likely to persist into the future [3]. The U.S. Office of Research Integrity (ORI) formally defines research misconduct as fabrication, falsification, or plagiarism in the process of proposing, conducting, reviewing, or reporting research [4]. In recent decades, increasing instances of misconduct—including data fabrication, plagiarism, and duplicate publication—have contributed to a sharp rise in article retractions. Since 1975, the number of retractions attributed to misconduct has increased more than tenfold [5, 6], undermining the credibility of scientific literature. Beyond damaging public trust, research misconduct wastes resources, impedes scientific progress, and discourages researchers from engaging with the scientific enterprise [7]. Studies suggest that the prevalence of scientific misconduct is particularly high in certain countries, including Iran, India, Turkey, South Korea, and China, with plagiarism being the most frequently reported form. One possible contributing factor is the linguistic challenge of writing and publishing in English, which may increase reliance on unethical shortcuts [6]. Moreover, institutional reluctance to acknowledge and address allegations—often manifesting as defensive or hostile reactions—can further contribute to the underreporting of misconduct [8].

Despite growing international awareness, relatively few studies have systematically examined research misconduct in developing countries, including Iran [9]. However, the limited available evidence from Iranian academic institutions reveals troubling patterns. Several studies have reported instances of fabrication, falsification, and plagiarism in both student theses and faculty publications [10–13]. One investigation found that 19% of undergraduate and 25% of postgraduate students admitted to engaging in at least one form of research misconduct [14]. Although the global academic community increasingly emphasizes research integrity, medical and health sciences students often lack adequate knowledge of ethical research principles [15]. Research ethics education remains critically underdeveloped in Iran's postgraduate programs. A national curriculum review revealed that 58% of 125 Master's and PhD programs provided no dedicated ethics instruction, and among the remainder, fewer than 14% offered structured standalone courses with defined content. This systemic training deficit provides essential context for our findings on research misconduct prevalence and underscores the urgent need for mandatory, standardized ethics education embedded within postgraduate medical curricula [16]. This is particularly concerning given the vital role these students play in generating and communicating health-related knowledge. If their research practices are ethically compromised, the dissemination of inaccurate or misleading findings may adversely affect public health outcomes.

Large international variations in self-reported misconduct rates—ranging from as low as 2% in the United Kingdom to as high as 99% in Croatia—highlight the influence of cultural, structural, and methodological differences in how misconduct is perceived and measured [17, 18]. Given the sensitivity of the topic and the likelihood of underreporting in direct surveys, indirect methods such as the Unmatched Count Technique (UCT) have been proposed as more effective tools for estimating the true prevalence of research misconduct. By comparing responses from groups that differ only in the inclusion of a sensitive item, UCT reduces social desirability bias and enhances response validity [19, 20].

This study aimed to estimate the prevalence of various forms of research misconduct among Iranian medical sciences students using UCT, a method designed to elicit more accurate responses on sensitive behaviors.

## Methods

### Study design and setting

This investigation adopted a cross-sectional approach in 2025, aimed at determining the prevalence of diverse research misconduct behaviors. The study focused on postgraduate students (at master's and doctoral levels) attending various medical universities nationwide in Iran. Eligibility required active enrollment in a postgraduate program and expressed interest in joining the research. However, non-Iranian students as well as Iranian students residing outside the country were excluded from the study.

Participants recruited from universities were located in 22 Iranian provinces—Tehran, Razavi and south Khorasan, Gilan, Hamadan, Qazvin, Kermanshah, Ilam, East and west Azerbaijan, Khuzestan, Kurdistan, Isfahan, and Sistan and Baluchestan, Chahar Mahal and Bakhtiari, Mazandaran, Fars, Arak, Ardebil, Yazd and Luristan, Kerman,—and were selected via convenience sampling based on the research team's pre-existing professional networks and collaborative relationships with researchers at these institutions.

### Sample size and sampling

The target sample size of *n* = 774 was calculated a priori using the standard formula for estimating a proportion, based on a reported prevalence of fabrication of 4.15% (Hadji et al.), with 95% confidence level and a 2% margin of error [11]. To compensate for anticipated non-response and incomplete submissions, we distributed the survey link to 1,060 eligible students. Ultimately, 730 participants provided complete responses, yielding a final analytical sample slightly below the calculated target.

Participant selection was based on convenience sampling. For assignment to the two variants of the questionnaire (Double List format), sequential random allocation was applied: one version was randomly selected and distributed until roughly half of the target sample was reached, and then the alternate version was then deployed to complete the sample.

### Data collection tool

This study utilized the Double-List Unmatched Count Technique (UCT). Two parallel lists (List A and List B) were designed, each comprising 12 sets of statements. In the first six sets of List A, each set includes four neutral (non-sensitive) statements. In List B, the same four neutral statements are repeated, but with the addition of one sensitive statement (related to research misconduct), making the set length five statements. In the second six sets of List A, each set includes four neutral statements plus one sensitive statement (length of five) and in List B, the same four neutral statements are repeated, but without the sensitive statement (length of four).

To construct these lists, we performed the following steps:

The initial step involved compiling a repository of 50 neutral statements by conducting structured online searches, examining prior literature, and organizing focus groups with postgraduate students.

Neutral statements were selected based on the following criteria:

1. No inherent sensitivity or near-sensitivity;
2. Strong conceptual precision without linguistic vagueness;
3. Mutual content autonomy among items;
4. Absence of linkage to the core sensitive study variable;
5. Neutrality across cultural and societal contexts;
6. Sufficient stability over time;
7. Capacity to yield equilibrated response patterns with anticipated rates near 50%.

#### Step 2: preliminary testing and filtering by response rates

The 50-statement repository was distributed to 30 postgraduate students selected through convenience methods, ensuring equal gender representation (50% female, 50% male).

Affirmative response frequencies were determined per statement. Those with rates under 30% or over 70% were discarded, while statements nearing 50% frequency were favored. This process sought to mitigate boundary effects and improve accuracy in gauging sensitive behavior occurrence.

#### Step 3: appraisal of face validity

Selected statements underwent a qualitative trial with 20 additional postgraduate students, gathering insights on phrasing clarity, ease of understanding, potential ambiguities, and expression smoothness. Adjustments were incorporated based on the input received.

#### Step 4: assessment of repeatability

After a two-week gap, the questionnaire was redistributed to the identical participants. The intraclass correlation coefficient (ICC) for the aggregate control item score was computed, with thresholds above 0.60 deemed satisfactory for reliability.

#### Step 5: refinement of sociodemographic elements

Following pilot participant suggestions, inquiries about academic discipline and institutional affiliation were omitted to bolster perceptions of confidentiality and alleviate identity disclosure fears. The final version retained solely age and gender as sociodemographic factors.

#### Configuration of double list UCT

Parallel sets of neutral statement were developed (List A and List B). The sensitive element (research misconduct behavior) was incorporated into one set per pair in a crossover design. Random group assignment placed participants into:

- Group 1: List A excluding the sensitive element + List B including the sensitive element (research misconduct behavior)
- Group 2: List A including the sensitive element (research misconduct behavior) + List B excluding the sensitive element

Respondents indicated only the count of applicable statements for each set.

The final version of questionnaire was implemented and administered via the Porsline online platform—a web-based survey system originating in Iran that supports the creation, dissemination, and statistical analysis of various digital forms and research instruments.

At the beginning of data collection, one of the two versions was randomly selected (sequential allocation) and distributed to eligible participants. This version remained active until the predetermined sample size for that List was fully achieved, after which the corresponding survey link was deactivated. Subsequently, the second questionnaire version was activated and distributed using the same recruitment channels until its target sample size was reached. To prevent duplicate submissions, IP restrictions were applied, allowing each individual to complete the questionnaire only once.

Recruitment was conducted through university collaborators from various Iranian cities. These collaborators disseminated the questionnaire link via student-oriented virtual social groups (such as Telegram or WhatsApp groups related to students). The recruitment timing spanned from March 2025 to September 2025.

Prior to accessing the survey, participants were presented with an electronic informed consent form. Progression to the questionnaire was contingent upon actively checking a mandatory box to confirm voluntary agreement to participate, thereby ensuring compliance with ethical research standards and safeguarding participant autonomy. In this study, no financial or material incentives were offered to participating students.

No personally identifiable information (such as names, identification numbers, email addresses, name of university and field of study or other traceable data) was collected.

### Instrument validity and reliability

The questionnaire was developed by the research team and underwent qualitative content validation and pilot testing. Thirty students assessed each item for clarity (simplification of ambiguous wording in items). The reported average clarity score of 75% reflects proportion of students who rated a specific item as "clear" or "very clear" to understand. Items considered unclear or potentially ambiguous were revised for improved clarity.

Test–retest reliability was evaluated over a two-week interval with 30 postgraduate students at Hamadan University of Medical Sciences, using the same list version at both administrations to assess temporal stability. The intraclass correlation coefficient [ICC (3,1), two-way mixed-effects model for absolute agreement] ranged from 0.64 (95% CI: 0.61–0.70) to 0.84 (95% CI: 0.80–0.88), indicating acceptable to good reliability. Although identical-item retesting may introduce practice effects, the two-week interval was selected to mitigate recollection bias while preserving construct stability.

The questionnaire used in this study, consisting of two separate list versions (List A and List B), is provided in the Appendix.

#### Quality control

The questionnaire was designed such that entering responses outside this range was impossible, and participants were required to answer each question to proceed to the next sections, thereby preventing missing data. To prevent duplicate submissions, IP restrictions were applied, allowing each individual to complete the questionnaire only once. Furthermore, no personal information (such as names or emails) was collected to ensure confidentiality, which simultaneously contributed to data quality by encouraging more honest responses. These overall measures were taken to minimize potential errors and enhance data validity, although completion time was not directly monitored.

### Statistical analysis

Responses were analyzed by comparing the average number of items endorsed between two groups: one group received a list that included the sensitive item, and the other group received a list containing only non-sensitive items. The difference between these two averages provides an estimate of the prevalence of the sensitive behavior. In the double-list design, each sensitive item was measured twice. Specifically, prevalence was estimated once using List A (sensitive list vs. its control list) and once using List B (sensitive list vs. its control list). The final prevalence estimate was calculated as the average of these two estimates.

$$\widehat{p}=\frac{1}{2}\left[({\overline{Y}}_{A+}-{\overline{Y}}_{A-})+({\overline{Y}}_{B+}-{\overline{Y}}_{B-})\right]$$

where ${\overline{Y} }_{A+}$ and ${\overline{Y} }_{B+}$ denote mean counts for sets containing the sensitive item in Lists A and B, respectively, and ${\overline{Y} }_{A-}$ and ${\overline{Y} }_{B-}$ denote mean counts for non-sensitive sets. The variance of the final estimate was obtained by adding the variances from both lists and subtracting their covariance. The standard error was then calculated by dividing this variance by the sample size and taking the square root. Using this standard error, a 95% confidence interval was constructed for each behavior [20, 21].

All statistical analyses were conducted using SPSS version 26. Results are reported as prevalence percentages with corresponding 95% confidence intervals.

## Results

A total of 730 postgraduate students from medical universities across Iran completed the survey, with 383 participants assigned to List A and 347 to List B. Imbalance in two list ensue from disruption in Iran. In fact, A planned second-wave assessment could not be completed due to extended university closures and internet disruptions stemming from regional security escalations in October 2023; consequently, participant engagement remained critically low even after institutions reopened three months later, precluding longitudinal analysis.

Table 1 presents the demographic characteristics by List. The mean age was similar between Lists (34.92 ± 6.78 years in List A vs. 35.21 ± 7.27 years in List B; independent t-test: t(728) = −0.559, p = 0.58), and gender distribution was approximately balanced (53.8% female in List A vs. 46.2% in List B; χ^2^ = 0.694, df = 1, p = 0.41), confirming homogeneity across Lists.

**Table 1:** Demographic characteristics by List

Characteristic	List A (*n* = 383)	List B (*n* = 347)	*P*-value)
Age, mean ± SD	34.92 ± 6.78	35.21 ± 7.27	0.58
Female, n (%)	206 (53.8%)	218 (46.2%)	0.41
Male, n (%)	177 (46.2%)	129 (53.8%)	

Floor and ceiling effects were examined by calculating the proportion of responses at the scale extremes (0 or 5) for each item. These proportions ranged from less than 1% (Item 7) to 13% (Item 2). Only Items 1 and 2 exhibited extreme-response rates exceeding 10%; all remaining items showed rates below 10%, with most clustering around 6%. This distribution suggests minimal floor or ceiling effects across the majority of items.

Table 2 presents the mean response per List, prevalence estimates (%), and 95% confidence intervals for each research misconduct.

**Table 2:** Prevalence of Research Misconduct by Item

Misconduct Behavior	List A Mean	List B Mean	Prevalence (%)	95% CI
Using others’ ideas without citation	0.64	0.22	43	[32,53]
Fabricated/manipulated/deleted data	0.27	0.4	34	[24,44]
Dishonest/selective reporting	0.34	0.42	38	[30,46]
Salami slicing	0.28	0.24	26	[15,37]
Purchased research work	0.2	0.2	20	[9,31]
Authorship misconduct	0.41	0.27	34	[24,45]

## Discussion

This study aimed to estimate the prevalence of research misconduct among postgraduate students at Iranian medical universities using the Unmatched Count Technique (UCT), an indirect survey method that minimizes social desirability bias and enhances honest reporting of sensitive behaviors. The results reveal that research misconduct is prevalent among this population, with the most common unethical behavior being the use of others’ ideas, phrases, or claims in research without proper citation or acknowledgment, reported by 43% of participants. Conversely, the least reported misconduct was purchasing part or all of a research project from someone else, admitted by 20%. Approximately one-third of misconduct cases involved data fabrication, manipulation, or intentional deletion, as well as unjustified authorship practices, both accounting for 34%. Additionally, “salami publication,” the practice of dividing research findings into multiple papers, was reported by 26%. These findings indicate that a substantial proportion of medical sciences students engage in a variety of unethical research practices. Our results partly align with previous studies, yet some important differences emerge. For example, Hofmann et al. (2013) reported that approximately 23% of Norwegian PhD students admitted to engaging in or witnessing scientific misconduct [22]. While their findings echo the presence of unethical practices, the lower prevalence compared to our study suggests possible influences of cultural, educational, and systemic factors that vary across countries. Similarly, a study conducted among 474 students in Switzerland and Germany employing the double list experiment methodology estimated moderate and severe plagiarism rates of 9% and 4%, respectively [19]. These figures contrast sharply with our 43% plagiarism estimate, likely reflecting differences in research culture, language proficiency, and enforcement of academic integrity policies.

A study investigating fabrication and falsification among researchers reported rates of 20% (95% CI: 8–32) and 39% (95% CI: 26–52), respectively [23], comparable to our findings of data manipulation at 34%. Furthermore, Hadji et al. found falsification and distortion rates of 4.15% and 12.65% using the randomized response technique, which are markedly lower than our results. This discrepancy may be partly explained by the narrower focus of their study on publication-related misconduct versus our broader examination encompassing multiple facets of research conduct across diverse medical disciplines.

Authorship misconduct—unjustified inclusion or exclusion of contributors—was reported by 34% of participants. This is consistent with findings by Wislar et al., who documented honorary authorship in 25% of original research papers and ghost authorship in 11.9% [24]. These issues underscore ongoing challenges in adhering to established authorship guidelines such as those recommended by the International Committee of Medical Journal Editors (ICMJE). Authorship misallocation compromises accountability and transparency, undermining the integrity of the scientific record.

The prevalence of salami publication in our study (26%) exceeds the 10–25% range reported in a Cochrane review focused on duplicate publication in biomedical research [25]. This difference likely results from our broader operational definition, which includes fragmented publication of partial findings without proper citation. Such practices dilute scientific contributions, inflate academic records, and burden the literature with redundant data.

Of particular concern is the finding that 20% of students admitted to purchasing research work. This unethical commodification threatens the fundamental principles of academic honesty and devalues the educational process. It is also indicative of systemic pressures that may compel students to seek shortcuts to meet academic requirements. The discrepancy may stem from our broader definition of duplicate publication, which includes repeated partial publications without proper citation. Teaching research ethics not only prevents misconduct but also fosters student interest in research. A study by Rostgar et al. found that 50% of medical students reported academic misconduct during clinical rotations, with stress and lack of accountability as key factors. Reducing stress, aligning education with student needs, and maintaining ongoing oversight may help mitigate such behaviors [26].

Our findings also support the critical role of ethics education in mitigating misconduct. Medical schools can reduce academic misconduct and promote student success by consistently enforcing academic integrity, supporting diverse student needs, and ensuring transparent curricula. Studies report misconduct rates in medical faculties ranging from 20 to 60% [27, 28]. These variations show how attitudes and behaviors are shaped by context, emphasizing the importance of targeted ethics education, which is key to raising awareness and preventing misconduct [29, 30]. In another study, cheating rates were lowest in midwifery (7.5%) and anesthesia (5.3%), while nursing students showed higher rates compared to other fields [31]. Similarly, another study reported higher cheating rates among nursing students [32]. Academic dishonesty and research misconduct present serious challenges to educational systems, potentially undermining the credibility of institutions, the quality of scientific outputs, and the competence of graduates. In the context of health education, such issues are even more critical [33].

Discrepancies in misconduct prevalence across countries reflect cultural, educational, and methodological differences. This variability underscores the importance of employing indirect, bias-minimizing methods such as the UCT to gain accurate estimates. Inadequate training in research ethics and divergent attitudes toward success undermine the culture of academic integrity. Given the active clinical roles of many health science students, unethical behaviors carry significant repercussions for both healthcare quality and public health outcomes. The complexity of health education, coupled with the lack of sustained ethics instruction, heightens the risk of misconduct. Therefore, academic institutions must integrate comprehensive, evidence-based ethics education, transparent policies, and continuous oversight into their curricula. Such measures are essential to cultivate a culture of integrity, accountability, and professionalism among future healthcare practitioners, ultimately enhancing scientific rigor and the quality of care.

### Strengths and limitations

This study’s strengths include the application of the UCT, which reduces social desirability bias common in direct questioning on sensitive topics, likely yielding more valid prevalence estimates. The relatively large sample size (n = 730) enhances the statistical power within the target population. Moreover, the investigation encompassed a broad range of misconduct types rather than focusing narrowly on plagiarism or fabrication, providing a more comprehensive picture of unethical research behaviors.

Although the Unmatched Count Technique (UCT) reduces social desirability bias in assessing sensitive behaviors, it relies on assumptions that cannot be fully empirically tested, and the absence of formal diagnostics for design effects constitutes an inherent methodological limitation. Data were collected through a voluntary online survey, introducing the possibility of self-selection bias. To enhance perceived anonymity, information on participants’ field of study and university affiliation was removed, which limited the assessment of representativeness and generalizability. In addition, sequential allocation to questionnaire versions may have resulted in residual imbalance between Lists. Finally, due to the online mode of data collection, likely potential interference among participants cannot be ruled out.

## Conclusion

The findings suggest that such behaviors may be present at non-negligible levels; however, they should be interpreted with caution in light of the methodological assumptions and limitations of the study. Nevertheless, the results highlight the importance of strengthening research ethics education, promoting transparent and enforceable institutional policies, and fostering a culture of academic integrity. Such measures may contribute to improving responsible research conduct and supporting the credibility of scientific outputs among future healthcare professionals.

## Supplementary Information

Supplementary Material 1.

## Data Availability

The corresponding author is responsible for the data. All relevant raw data will be freely available to any interested researcher.

## Abbreviations

UCT: Unmatched Count Technique
ICC: Intraclass Correlation Coefficient
