# Supplementary material for: Indirect measurement of research misconduct among Iranian postgraduate students of medical sciences using the unmatched count technique
Source: Res Integr Peer Rev. 2026 Jun 28;11:27. doi: 10.1186/s41073-026-00202-5 (PMC13310431; doi:10.1186/s41073-026-00202-5)
Supplement: Supplementary file 1 — Supplementary Material 1. [file 41073_2026_202_MOESM1_ESM.docx]

Appendix. Questionnaire **(List A)**

This questionnaire, which was translated into English for reporting purposes, was administered online via the Porsline platform and used to estimate the prevalence of six research misconduct behaviors among postgraduate students at Iranian medical sciences universities using the unmatched count technique (UCT).

Set 1

Please indicate how many of the following statements apply to you:

1. The last digit of my mobile phone number is odd.

2. I prefer hot weather to cold weather.

3. I usually prefer traveling by train over airplane.

4. I was born in the first half of the year.

Number of statements that apply to you:

☐ 0 ☐ 1 ☐ 2 ☐ 3 ☐ 4

Set 2

Please indicate how many of the following statements apply to you:

1. I order take-out food at least once per month.

2. I prefer pop music to traditional music.

3. I engage in at least one hour of walking per week.

4. If a pedestrian overpass is available, I use it to cross the street.

Number of statements that apply to you:

☐ 0 ☐ 1 ☐ 2 ☐ 3 ☐ 4

Set 3

Please indicate how many of the following statements apply to you:

1. I prefer rainy weather to sunny weather.

2. I prefer wearing sports shoes to formal shoes.

3. I am generally an extroverted person.

4. I prefer sour foods to sweet foods.

Number of statements that apply to you:

☐ 0 ☐ 1 ☐ 2 ☐ 3 ☐ 4

Set 4

Please indicate how many of the following statements apply to you:

1. Persepolis football team is one of my favorite teams.

2. The first digit of my national ID number is even.

3. I enjoy cooking.

4. I am interested in historical books.

Number of statements that apply to you:

☐ 0 ☐ 1 ☐ 2 ☐ 3 ☐ 4

Set 5

Please indicate how many of the following statements apply to you:

1. I am interested in fashion and clothing.

2. I listen to music for at least one hour every day.

3. I enjoy watching football.

4. I am interested in science-fiction films.

Number of statements that apply to you:

☐ 0 ☐ 1 ☐ 2 ☐ 3 ☐ 4

Set 6

Please indicate how many of the following statements apply to you:

1. I visit a barber/hairdresser at least once per month.

2. I tend to stay up late at night.

3. I prefer doogh (a yogurt-based drink) to soft drinks.

4. The last digit of my national ID number is even.

Number of statements that apply to you:

☐ 0 ☐ 1 ☐ 2 ☐ 3 ☐ 4

Set 7

Please indicate how many of the following statements apply to you:

1. The last digit of my national ID number is odd.

2. I prefer traditional music to pop music.

3. I usually prefer traveling by airplane over train.

4. I have, at least once, added the name of an individual who made no contribution to the research as a co-author.

5. If a pedestrian overpass is available, I always use it.

Number of statements that apply to you:

☐ 0 ☐ 1 ☐ 2 ☐ 3 ☐ 4 ☐ 5

Set 8

Please indicate how many of the following statements apply to you:

1. I do not order take-out food at least once per month.

2. I have, at least once, purchased part or all of a research project from another person.

3. I enjoy watching football.

4. I go to bed before 10 p.m. most nights.

5. I prefer sour foods to sweet foods.

Number of statements that apply to you:

☐ 0 ☐ 1 ☐ 2 ☐ 3 ☐ 4 ☐ 5

Set 9

Please indicate how many of the following statements apply to you:

1. I am not interested in fashion and clothing.

2. I usually prefer formal shoes to sports shoes.

3. Esteghlal football team is one of my favorite teams.

4. I have, at least once, divided the results of a single study into multiple publications in order to increase the number of published articles.

5. I was born in the second half of the year.

Number of statements that apply to you:

☐ 0 ☐ 1 ☐ 2 ☐ 3 ☐ 4 ☐ 5

Set 10

Please indicate how many of the following statements apply to you:

1. I am not interested in science-fiction films.

2. I visit a barber/hairdresser at least once per month.

3. I usually go to bed early at night.

4. I have, at least once, reported or used non-existent data in my research work.

5. I do not prefer doogh to soft drinks.

Number of statements that apply to you:

☐ 0 ☐ 1 ☐ 2 ☐ 3 ☐ 4 ☐ 5

Set 11

Please indicate how many of the following statements apply to you:

1. I do not prefer hot weather to cold weather.

2. I have, at least once, altered or manipulated data in my research to obtain desired results.

3. I am not interested in historical books.

4. I usually listen to music for one hour per day.

5. The first digit of my national ID number is odd.

Number of statements that apply to you:

☐ 0 ☐ 1 ☐ 2 ☐ 3 ☐ 4 ☐ 5

Set 12

Please indicate how many of the following statements apply to you:

1. Persepolis football team is not one of my favorite teams.

2. I have, at least once, fabricated data in my research work.

3. I prefer wearing sports shoes to formal shoes.

4. The last digit of my mobile phone number is even.

5. I engage in at least one hour of walking per week.

Number of statements that apply to you:

☐ 0 ☐ 1 ☐ 2 ☐ 3 ☐ 4 ☐ 5

Appendix. Questionnaire **(List B)**

This questionnaire, which was translated into English for reporting purposes, was administered online via the Porsline platform and used to estimate the prevalence of six research misconduct behaviors among postgraduate students at Iranian medical sciences universities using the unmatched count technique (UCT).

Set 1

Please indicate how many of the following statements apply to you:

1. The last digit of my mobile phone number is odd.

2. I prefer hot weather to cold weather.

3. I usually prefer traveling by train over airplane.

4. I was born in the first half of the year.

5. I have, at least once, used ideas, phrases, or claims from others in my research work without proper citation or acknowledgment.

Number of statements that apply to you:

☐ 0 ☐ 1 ☐ 2 ☐ 3 ☐ 4 ☐ 5

Set 2

Please indicate how many of the following statements apply to you:

1. I order take-out food at least once per month.

2. I have, at least once, fabricated data or reported non-existent results in my research work.

3. I prefer pop music to traditional music.

4. I engage in at least one hour of walking per week.

5. If a pedestrian overpass is available, I use it to cross the street.

Number of statements that apply to you:

☐ 0 ☐ 1 ☐ 2 ☐ 3 ☐ 4 ☐ 5

Set 3

Please indicate how many of the following statements apply to you:

1. I prefer rainy weather to sunny weather.

2. I prefer wearing sports shoes to formal shoes.

3. I am generally an extroverted person.

4. I prefer sour foods to sweet foods.

5. I have, at least once, manipulated or altered my data or findings in research work in order to increase the likelihood of publication.

Number of statements that apply to you:

☐ 0 ☐ 1 ☐ 2 ☐ 3 ☐ 4 ☐ 5

Set 4

Please indicate how many of the following statements apply to you:

1. Persepolis football team is one of my favorite teams.

2. The first digit of my national ID number is even.

3. I enjoy cooking.

4. I have, at least once, divided the results of a single research project into multiple publications (each containing less than 50% of the original findings) solely to increase the number of published articles.

5. I am interested in historical books.

Number of statements that apply to you:

☐ 0 ☐ 1 ☐ 2 ☐ 3 ☐ 4 ☐ 5

Set 5

Please indicate how many of the following statements apply to you:

1. I am interested in fashion and clothing.

2. I listen to music for at least one hour every day.

3. I have, at least once, added a person as co-author who made no contribution to the research, or removed a person who did contribute.

4. I enjoy watching football.

5. I am interested in science-fiction films.

Number of statements that apply to you:

☐ 0 ☐ 1 ☐ 2 ☐ 3 ☐ 4 ☐ 5

Set 6

Please indicate how many of the following statements apply to you:

1. I visit a barber/hairdresser at least once per month.

2. I have, at least once, purchased all or part of a research project from another person.

3. I tend to stay up late at night.

4. I prefer doogh (a yogurt-based drink) to soft drinks.

5. The last digit of my national ID number is even.

Number of statements that apply to you:

☐ 0 ☐ 1 ☐ 2 ☐ 3 ☐ 4 ☐ 5

Set 7

1. The last digit of my national ID number is odd.

2. I prefer traditional music to pop music.

3. I usually prefer traveling by airplane over train.

4. If a pedestrian overpass is available, I always use it.

Number of statements that apply to you:

☐ 0 ☐ 1 ☐ 2 ☐ 3 ☐ 4

Set 8

1. I do not order take-out food at least once per month.

2. I enjoy watching football.

3. I go to bed before 10 p.m. most nights.

4. I prefer sour foods to sweet foods.

Number of statements that apply to you:

☐ 0 ☐ 1 ☐ 2 ☐ 3 ☐ 4

Set 9

1. I am not interested in fashion and clothing.

2. I usually prefer formal shoes to sports shoes.

3. Esteghlal football team is one of my favorite teams.

4. I was born in the second half of the year.

Number of statements that apply to you:

☐ 0 ☐ 1 ☐ 2 ☐ 3 ☐ 4

Set 10

1. I am not interested in science-fiction films.

2. I visit a barber/hairdresser at least once per month.

3. I usually go to bed early at night.

4. I do not prefer doogh to soft drinks.

Number of statements that apply to you:

☐ 0 ☐ 1 ☐ 2 ☐ 3 ☐ 4

Set 11

1. I do not prefer hot weather to cold weather.

2. I am not interested in historical books.

3. I usually listen to music for one hour per day.

4. The first digit of my national ID number is odd.

Number of statements that apply to you:

☐ 0 ☐ 1 ☐ 2 ☐ 3 ☐ 4

Set 12

1. Persepolis football team is not one of my favorite teams.

2. I prefer wearing sports shoes to formal shoes.

3. The last digit of my mobile phone number is even.

4. I engage in at least one hour of walking per week.

Number of statements that apply to you:

☐ 0 ☐ 1 ☐ 2 ☐ 3 ☐ 4
